# Icariin prevents oestrogen deficiency–induced alveolar bone loss through promoting osteogenesis via STAT3

**DOI:** 10.1111/cpr.12743

**Published:** 2020-01-13

**Authors:** Hongyuan Xu, Siru Zhou, Ranyi Qu, Yiling Yang, Xinyi Gong, Yueyang Hong, Anting Jin, Xiangru Huang, Qinggang Dai, Lingyong Jiang

**Affiliations:** ^1^ Center of Craniofacial Orthodontics Department of Oral and Cranio‐maxillofacial Science Ninth People's Hospital Shanghai Jiaotong University School of Medicine Shanghai Key Laboratory of Stomatology & Shanghai Research Institute of Stomatology National Clinical Research Center of Stomatology Shanghai China; ^2^ The 2nd Dental Center Ninth People's Hospital Shanghai Jiaotong University School of Medicine Shanghai Key Laboratory of Stomatology & Shanghai Research Institute of Stomatology National Clinical Research Center of Stomatology Shanghai China

**Keywords:** alveolar bone osteoporosis, Icariin, mandibular bone marrow stromal cell, osteogenesis, STAT3

## Abstract

**Objectives:**

Alveolar bone osteoporosis has attracted more and more attention because of its profound impact on stomatognathic function and treatment, but current treatments have not been targeted to alveolar bone and might even cause severe side effects. Thus, identifying the effects of anti‐osteoporosis agents on alveolar bone is essential. Icariin ameliorates metabolic dysfunction of long bones, but its effects on alveolar bone remain unclarified.

**Materials and methods:**

BMSCs were isolated from rat mandibles (mBMSCs). The osteogenic potential of mBMSCs and the signalling pathway involved under icariin treatment were measured by ALP and alizarin red staining, reverse transcription‐polymerase chain reaction (RT‐PCR), Western blotting and immunofluorescence. Dual‐luciferase assay, chromatin immunoprecipitation (ChIP) and co‐immunoprecipitation were used to investigate the molecular mechanism. Ovariectomized and sham‐operated rats treated with or without icariin were analysed by micro‐CT, TRAP staining and calcein double labelling.

**Results:**

We found that icariin promoted osteoblast differentiation of mBMSCs. Furthermore, STAT3 was critical for icariin‐promoted osteoblast differentiation, as indicated by increased phosphorylation levels in icariin‐treated mBMSCs, while preventing STAT3 activation blocked icariin‐induced osteoblast differentiation. Mechanistically, icariin‐promoted transcription of the downstream osteogenic gene *osteocalcin* (*Ocn*) through STAT3 and STAT3 bound to the promoter of *Ocn*. Notably, icariin prevented the alveolar bone osteoporosis induced by oestrogen deficiency through promoting bone formation.

**Conclusions:**

For the first time, our work provides evidence supporting the potential application of icariin in promoting osteogenesis and treating alveolar bone osteoporosis.

## 1 INTRODUCTION

With the worldwide problem of the ageing population, increasing numbers of elderly patients require dental treatment. Because of the high rate of osteoporosis in aged patients, especially post‐menopausal osteoporosis in elderly women,^1^ the effects of osteoporosis on oral health have become the focus of both clinical dentistry and basic research. Alveolar bone is an irregular protuberance of the jawbone that accommodates and provides adequate support for the teeth. A series of studies have indicated a decrease in alveolar bone density and quality in women with post‐menopausal osteoporosis.^2^, ^3^, ^4^, ^5^ Our previous studies also indicated that oestrogen deficiency induces alveolar bone loss and trabecular fragmentation in rats.^6^ Oestrogen deficiency–induced alveolar bone loss may cause some adverse impacts on periodontal^7^ and orthodontic treatment,^8^ even causing tooth loss.^9^ Thus, how to prevent and treat alveolar bone loss has become an important issue.

Post‐menopausal osteoporosis is the result of bone resorption exceeding bone formation and is induced by oestrogen deficiency.^10^ Bisphosphonates, which inhibit osteoclast differentiation and bone resorption activity, are used as the only first‐line prescription treatment for osteoporosis.^11^ Despite their remarkable effects on bone volume, concerns about side effects such as atypical femur fractures and osteonecrosis of the jaw are leading many patients to refuse these drugs, especially those who also need dental treatment.^12^, ^13^ Therefore, it remains an important clinical need to develop prevention and treatment strategies for oestrogen deficiency–induced alveolar bone loss.

In the very first studies into post‐menopausal osteoporosis, researchers found that oestrogen was effective in both prevention^14^ and treatment^15^ of bone loss induced by oestrogen deficiency. However, because of side effects including an increase in cardiovascular events and breast cancer risk, oestrogen replacement therapy was not recommended for long‐term use for osteoporosis.^16^ Recently, there has been increasing interest in the use of phytoestrogens as substitutes for oestrogen due to their structural and functional similarities but absence of serious side effects.^17^, ^18^ Herba Epimedii, one of the most active phytoestrogens,^19^, ^20^, ^21^ is a traditional Chinese medicine that has been commonly used as an anti‐rheumatic and anti‐atherosclerotic agent in China. Icariin (C_33_H_40_O_15_, ICA) is the major active ingredient of Herba Epimedii and has been proven to be beneficial for bone metabolism in the long bone system.^22^ Icariin has been reported to protect against bone loss induced by oestrogen deficiency.^23^, ^24^ Alveolar bone exhibits differences in morphologic features, metabolic rate and pharmaceutical reactions to the long bone system.^25^, ^26^ Therefore, it is very necessary to clarify the effects of icariin on alveolar bone and to elucidate its underlying mechanisms.

Multiple mechanisms and targets have been reported for the therapeutic effects of icariin on autoimmune diseases such as rheumatoid arthritis and bronchial asthma.^27^ These include the NF‐κB and Erk‐p38‐JNK signalling pathways, as well as other targets such as TLRs and STATs. The signal transducer and activator of transcription (STAT) protein family consists of seven members: STAT1, STAT2, STAT3, STAT4, STAT5A, STAT5B and STAT6. STAT proteins play a fundamental role in many cellular processes, including cell growth and differentiation, apoptosis, immune responses and inflammation, which can be activated through phosphorylation in response to various cytokines and growth factors.^28^ STAT3 is expressed in osteoblasts, osteoclasts and osteocytes.^29^ Studies have shown that dominant‐negative mutations in the human STAT3 gene result in osteoporosis and pathologic fractures.^30^, ^31^ A growing body of evidence suggests that STAT3 plays essential roles in skeletal metabolism through regulating anabolic signals in osteoblasts.^32^ Hence, we hypothesize that STAT3 may play an important role in icariin‐mediated osteoblast differentiation and bone metabolism.

The present study investigated the effects of icariin on alveolar bone and its underlying mechanisms in vitro and in vivo with the aim of laying the foundation for application of icariin in the treatment of alveolar bone metabolic disorders.

## 2 MATERIALS AND METHODS

### 2.1 Harvest and culture of rat mandibular BMSCs

**Figure 1:**
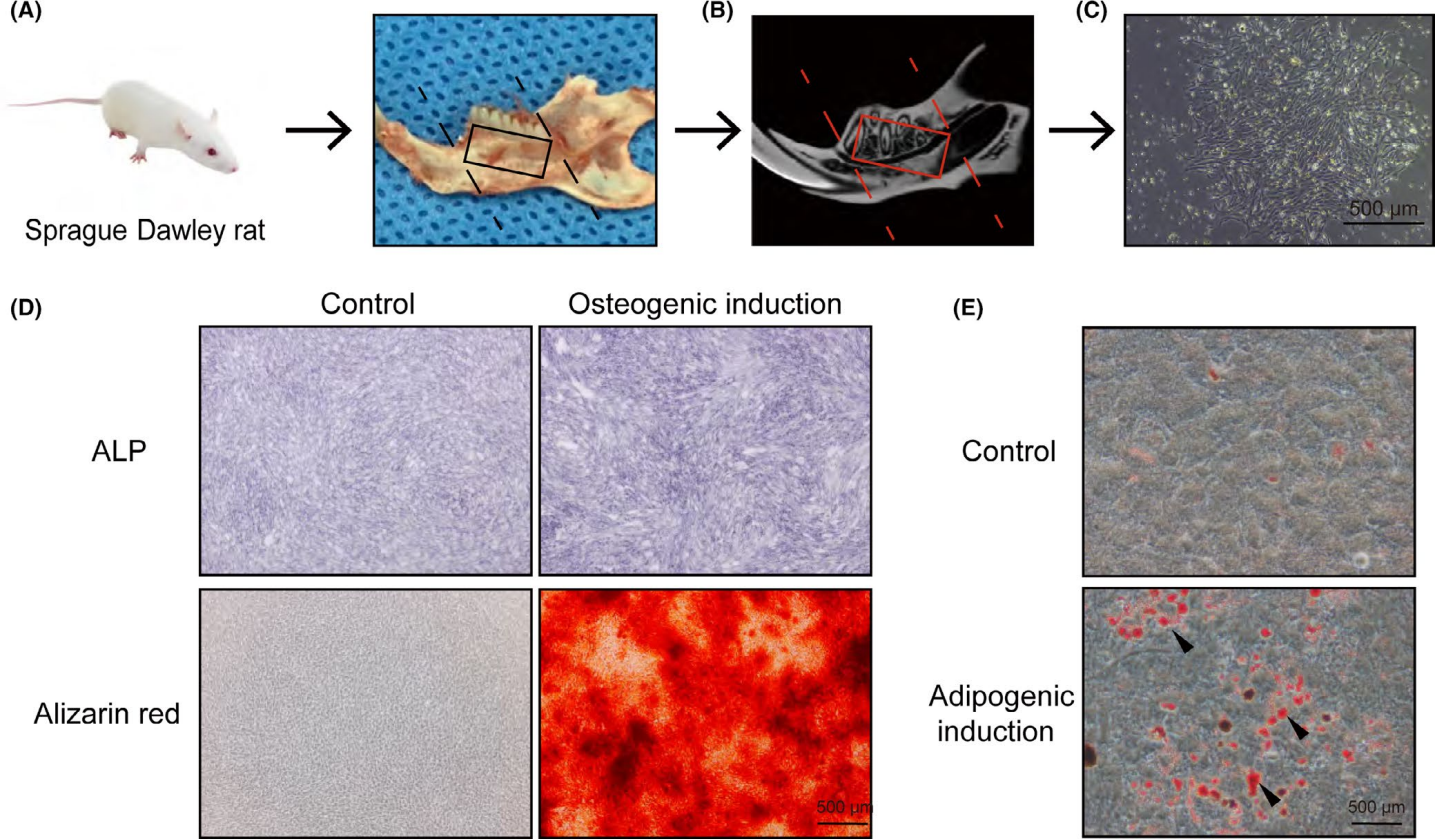
Isolation and culture of mandibular BMSCs (mBMSCs). A, Separation of the mandible from the rest of the body by soft tissue dissection. B, A representative micro‐CT image in the sagittal view showing the marrow space and the porous low‐density bone within the mandible. C, Brightfield microscopic image of mBMSCs of passage 0 at day 6. D, Microscopic observation of mBMSCs after ALP staining and alizarin red staining. E, Microscopic observation of mBMSCs after Oil Red O staining

After separation of the mandible, the incisors were disconnected along the posterior edge of the first molar and the mandibular ascending ramus was removed along the posterior edge of the third molar to expose the trabecular bone (Figure 1A and B). Using a 10 mL needle, the bone marrow was flushed with α‐MEM (HyClone). Cells were harvested and pooled in a 10 cm dish. They were cultured in α‐MEM with 10% foetal bovine serum (HyClone), 1% penicillin and 1% streptomycin (Gibco, Thermo Fisher Scientific) at 37°C in a humidified incubator containing 5% CO_2_. Every 3 days, the medium was refreshed until the cells reached 70%‐80% confluence. After that, the cells were passaged or seeded into plates.

To induce osteoblastic differentiation, cells were seeded into six‐well plates at a density of 1.0 × 10^5^ cells per well and fed with osteogenic induction medium containing 100 nmol/L dexamethasone, 50 μmol/L ascorbic acid and 10 mmol/L β‐glycerophosphate (all from Sigma‐Aldrich). After 7 or 14 days of induction, cells were analysed by alkaline phosphatase (ALP) staining and alizarin red staining.

To induce adipogenic differentiation, cells were seeded into six‐well plates at a density of 2.0 × 10^5^ cells per well with adipogenic induction medium containing 10 μg/mL insulin, 1 μmol/L dexamethasone, 200 μmol/L indomethacin and 500 μmol/L 3‐isobutyl‐1‐methylxanthine. After 2 days of induction, cells were switched to maintenance medium containing 10 μg/mL insulin for 1 day. After further alternation of differentiation and maintenance conditions for 14 days, cells were analysed by Oil Red O staining (Cyagen US Inc, RASMX‐90031).

### 2.2 Alkaline phosphatase and alizarin red staining

Cells were washed with PBS three times and fixed with 4% paraformaldehyde at room temperature for 15 minutes. Cells were then incubated in the dark at 37°C with ALP staining working solution, following the common protocol.

For alizarin red staining, cells were rinsed with ice‐cold PBS three times and fixed with 4% paraformaldehyde at room temperature for 15 minutes. Cells were stained with 40 mmol/L alizarin red S (Sigma‐Aldrich), pH 4.2, for 15 minutes with gentle shaking.

### 2.3 Oil red O staining

Cells were washed with PBS three times, fixed with 4% paraformaldehyde at room temperature for 15 minutes, and then washed with distilled water and air‐dried. Freshly filtered 60% Oil Red O working solution was added and incubated for 1 hour at room temperature, after which cells were washed with 70% ethanol before red‐stained lipid droplets were observed under a light microscope.

### 2.4 Cell proliferation assay

After cells reached 80% confluence, cultures were treated with 0, 10, 20, 40, 80 or 160 μmol/L icariin according to previous reports^33^, ^34^ (Tauto Biotech Company), with 1 μL/mL DMSO as control. Cell survival was separately evaluated using a CCK8 kit (Dojindo Molecular Technologies Inc). All experiments were repeated at least three times.

### 2.5 Apoptosis assay

To assess the effects on apoptosis of mandibular BMSCs, we used an Annexin V‐FITC Apoptosis Detection Kit II (BD Pharmingen). Cells were analysed using the FACSCalibur system (BD Biosciences).

### 2.6 Chromatin immunoprecipitation and real‐time reverse transcription‐polymerase chain reaction analysis

Chromatin immunoprecipitation analysis in C_3_H_10_ cells was performed using an Enzymatic Chromatin Immunoprecipitation Kit (EZ ChIPTM #17‐371, Merck‐Millipore) following the manufacturers' instructions. Briefly, C_3_H_10_ cells were cross‐linked with 1% formaldehyde for 10 minutes at room temperature followed by quenching with glycine. Chromatin digestion was performed by micrococcal nuclease to obtain DNA fragments from 150 bp to 900 bp. Immunoprecipitation was performed with STAT3 (#1264, Cell Signaling Technology), and IgG was used as a negative control. Precipitated DNA was detected by qPCR with specific primers. Primers for the STAT3 binding site in the *Ocn* promoter were 5′GGATACCCCATGTTCCCAGC3′ and 5′TGCAGCCCGTCTACTGGAGC3′.

Real‐time PCR was conducted with a Roche LC 480 system using SYBR1 Premix (TaKaRa Bio Inc) on the basis of the manufacturer's instructions. All samples were analysed in triplicate, and β‐actin was used as an internal control. The primer sequences used in this study are listed in Table 1.

**Table 1:** Primers used and their representative sequences

GENES	Forward and reverse sequences
*β‐Actin*	5′CCCATACCCACCATCACACC 5′CACCCGCGAGTACAACCTTC
*Alkaline phosphatase* (*Alp*)	5′CACTAGCAAGAAGAAGCCTTTGG 5′TATGTCTGGAACCGCACTGAAC
*Runt‐related transcription factor 2*(*Runx2*)	5′GGGACCATTGGGAACTGATAGG 5′ATCCAGCCACCTTCACTTACACC
*Osterix*(*Osx*)	5′GGTAGGAGTGTTGCCAGGAC 5′CTGACTGCCTGCCTAGTGTC
*Collagen type I* (*Col I*)	5′CAGGCTGGTGTGATGGGATT 5′CCAAGGTCTCCAGGAACACC
*Bone sialoprotein*(*Bsp*)	5′CCAGAAAGAGCAGCACGGTTGAG 5′TGACCCTCGTAGCCTTCATAGCC
*Osteopontin*(*Opn*)	5′CCAGCCAAGGACCAACTACA 5′AGTGTTTGCTGTAATGCGCC
*Osteocalcin* (*Ocn*)	5′GAATAGACTCCGGCGCTACC 5′AGCTCGTCACAATTGGGGTT

### 2.7 Western blotting and co‐immunoprecipitation

Cells were lysed with RIPA lysis buffer containing protease inhibitors (Bocai). Equivalent amounts of protein were subjected to 10% sodium dodecyl sulphate‐polyacrylamide gel electrophoresis (SDS‐PAGE) (Bio‐Rad). After that, the proteins were electro‐transferred onto nitrocellulose blotting membranes (GE Healthcare) and then blocked using 5% skimmed milk (BD Biosciences) for 1 hour. After blocking, the membranes were washed and incubated with primary antibodies at 4°C overnight and then incubated with the HRP‐linked IgG secondary antibody (1:1000, Beyotime Institute of Biotechnology). The primary antibodies used were as follows: Runx2 (Santa Cruz Biotechnology; sc‐390351), p‐STAT3 (Cell Signaling Technology [CST]; 9145), STAT3 (sc‐8019) and GAPDH (CST; 5174). The protein bands were visualized using an enhanced chemiluminescence detection system (Millipore).

Co‐IP was conducted according to a procedure previously described.^35^ Briefly, 293T cells were seeded into a 10 cm dish at a density of 3 × 10^6^ cells. After 24 hours, Flag‐STAT3 and HA‐Runx2 expression plasmids were transfected with Lipofectamine 2000 (Life Technologies) according to a previously reported protocol.^36^ After a further 48 hours, cells were lysed and whole cell lysates were used for immunoprecipitation by Flag antibody (Sigma‐Aldrich) at 4°C overnight. Western blotting was then performed with the indicated antibodies (anti‐HA and anti‐Flag, Sigma‐Aldrich).

### 2.8 Immunofluorescence

mBMSCs from 4‐week‐old rats were fixed with 4% PFA for 30 minutes at room temperature. After being washed in PBS, cells were permeabilized with 0.2% Triton X‐100 and blocked with 5% BSA for 60 minutes. Samples were incubated with p‐STAT3 antibodies and STAT3 antibodies overnight at 4°C. Next day, the samples were washed and incubated with goat anti‐rabbit Alexa Fluor 594 (33112ES60, YEASEN) and goat anti‐mouse Alexa flour 488 (33206ES60, YEASEN) as secondary antibody for 1 hour at room temperature, and then counterstained with DAPI (D8417, Sigma‐Aldrich). Images were captured under a fluorescence microscope (IX83, Olympus).

### 2.9 Plasmids

cDNA of *Stat3* was cloned into a phage‐based plasmid. The Runx2 plasmid was a gift from Dr Gerard Karsenty's laboratory. The *Ocn* promoter‐driven pGL3‐based luciferase reporter was synthesized.

### 2.10 Transient transfection and luciferase assay

HEK 293T cells were seeded into 24‐well plates, then transfected with an *Ocn* promoter‐driven pGL3‐based luciferase reporter gene plasmid and varied combinations of Flag‐STAT3 and HA‐Runx2 plasmids using Lipofectamine 2000. pRL‐TK (Promega) was co‐transfected as a normalization control for transfection efficiency. Cells were treated with varied combinations of icariin and the inhibitor of upstream phosphorylases AG490. After 48 hours, cells were lysed with lysis buffer and the supernatants were used for dual‐luciferase reporter assay (Promega) according to the manufacturer's instructions. Luminescent signals normalized to firefly luciferase were used to represent reporter activity.

### 2.11 Animals and treatment procedure

All animal experimental procedures conducted in this study were approved by the Animal Care Committee of Shanghai Ninth People's Hospital, Shanghai Jiao Tong University School of Medicine. Female Sprague Dawley (SD) rats (Shanghai SLAC Laboratory Animal Co. Ltd) were purchased at the age of 4 weeks and kept in a temperature‐ and humidity‐controlled room (23 ± 3°C and 60% ± 5%, respectively) with a 12‐hour light/dark cycle under specific pathogen‐free (SPF) conditions. Forty‐five female Sprague Dawley rats aged 8 weeks old were randomly allocated into three groups: (a) fifteen animals were sham‐operated; (b) fifteen animals underwent surgical ovariectomy (OVX): bilaterally ovariectomized; and (c) fifteen animals underwent surgical ovariectomy were intraperitoneally injected with icariin once every day at 20 mg/kg.

### 2.12 Micro‐CT scanning and alveolar bone analysis

At 3 months after ovariectomy, rats were sacrificed under 10% chloral hydrate anaesthesia and maxillae were collected. Both sides of the maxillae were collected from the body and fixed in 4% paraformaldehyde. Samples were scanned using a micro‐CT scanner (Scanco μCT 80, Scanco Medical AG, Bassersdorf, Switzerland) with a 16 μm voxel size. The density of maxilla specimens was standardized to that of hydroxyapatite, and software affiliated to the micro‐CT scanner was used to reconstruct its 3D structure. For alveolar bone, the region of interest (ROI) was chosen in the inter‐radicular region of the right maxillary first molar, keeping away from the roots. The following structural parameters of the ROI were calculated: bone mineral density (BMD), bone volume/tissue volume (BV/TV), trabecular number (Tb.N), trabecular thickness (Tb.Th) and trabecular separation (Tb.Sp).

### 2.13 Histological analysis of alveolar bone

At one month after ovariectomy, rats were sacrificed under 10% chloral hydrate anaesthesia and maxillae were collected. Samples were fixed in 4% paraformaldehyde for 48 hours, followed by decalcification for approximately 4 weeks with 15% ethylenediaminetetraacetic acid (EDTA) and then embedded in paraffin. Sections were prepared along the plane parallel to the long axis of the tooth and then cut into 4 μm thick serial sagittal sections. Tartrate‐resistant acid phosphatase (TRAP) staining was used to detect osteoclasts according to the instructions with an acid phosphatase leukocyte kit (Sigma‐Aldrich).

### 2.14 Calcein‐alizarin red double labelling

At 3 weeks after ovariectomy, rats received intraperitoneal injection of 20 mg/kg calcein (CA, 1 mg/mL in 2% NaHCO_3_ solution) on day 0 and 40 mg/kg alizarin red S (AL, 2 mg/mL in H_2_O) on day 4. Rats were sacrificed on day 7, and isolated maxillae were dehydrated and embedded in polymethylmethacrylate. Samples were cut into 5 μm sections with a hard tissue cutter (RM2265, Leica), and fluorescence‐labelled images were captured using a microscope (BX51, Olympus). The bone formation activity represented by mineral apposition rate (MAR) was measured according to standard methods.

### 2.15 Statistical analysis

All quantitative data are shown as the mean ± SD. For two‐group comparison, Student's *t* test was used for evaluations. For comparison among more than two groups, one‐way analysis of variance (ANOVA) was used for evaluation. Values of *P* < .05 were considered significant.

## 3 RESULTS

### 3.1 Mandibular BMSC isolation and identification

Passage‐0 (P0) mBMSCs isolated from mandibular bone marrow showed well‐spread attachment and homogeneous spindle morphology with a radial colony arrangement (Figure 1C). Primary mBMSCs exhibited multi‐directional differentiation, as shown by increased ALP activity, mineralized matrix production and adipocyte formation after osteogenic and adipogenic induction (Figure 1D and E).

### 3.2 Effects of icariin on proliferation of mBMSCs

**Figure 2:**
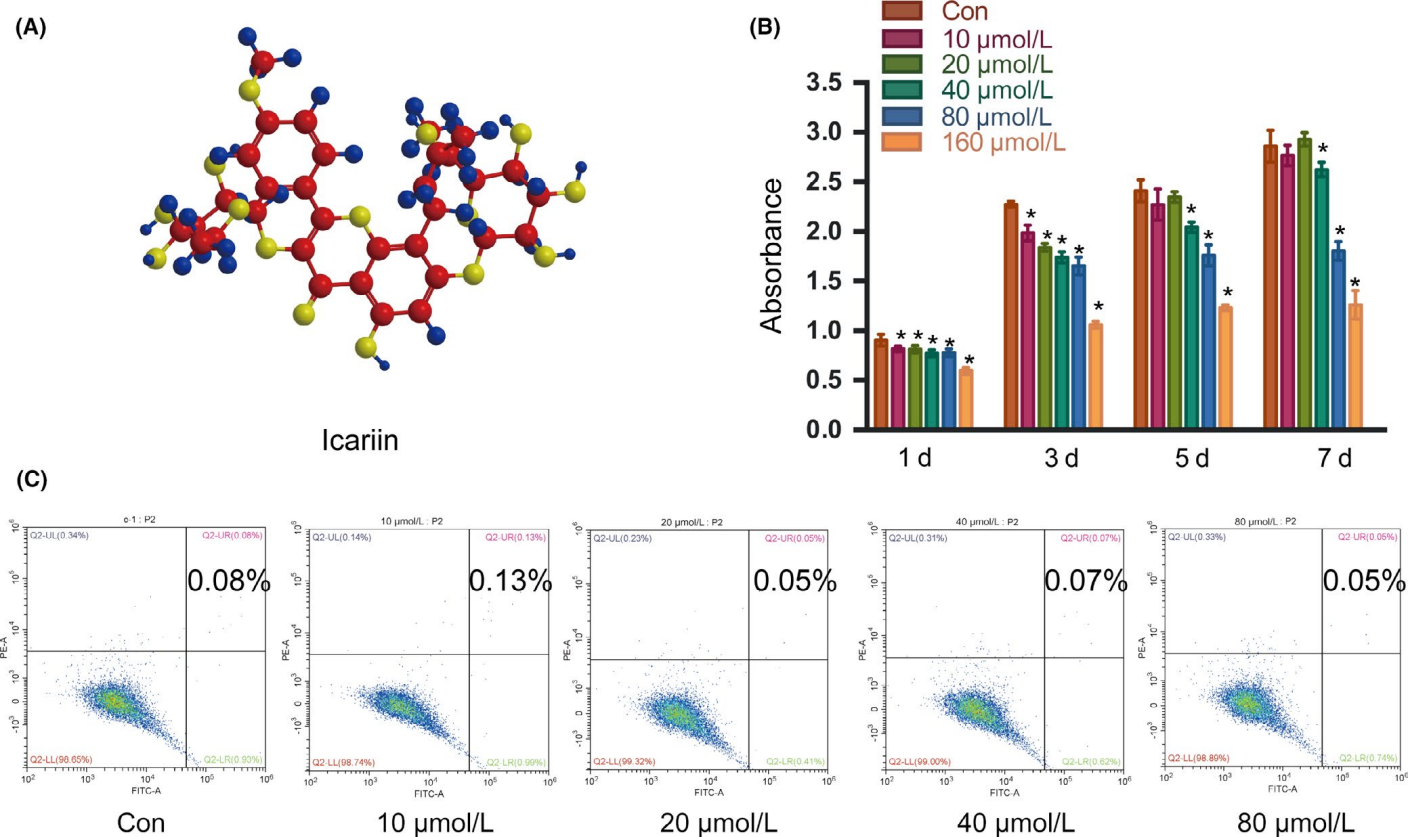
Effects of icariin on the proliferation and apoptosis of mBMSCs. A, Structure of icariin. B, Proliferation of mBMSCs with different concentrations of icariin, analysed by CCK‐8 assay. C, Apoptosis of mBMSCs with different concentrations of icariin. Error bars represent mean ± SD. *P < .05 compared with control group, n = 5

In order to clarify the effects of icariin (Figure 2A) on mBMSCs, we first investigated the effects of different concentrations of icariin on cell proliferation and apoptosis. CCK8 assay was used to determine the effects on the growth curve of mBMSCs. As shown in Figure 2B, high doses of icariin (40, 80, or 160 μmol/L) resulted in a compromised cell proliferation rate in a concentration‐dependent manner, while there were no differences between controls and cells treated with lower concentrations (10 or 20 μmol/L). Furthermore, apoptosis assay of BMSCs after 3 days of icariin treatment showed no effect of icariin on apoptosis of mBMSCs at any concentration (Figure 2C). Thus, 10 and 20 μmol/L icariin was used in subsequent experiments to investigate the effects of icariin on osteogenesis of BMSCs and to explore the molecular mechanisms involved.

### 3.3 Icariin promotes osteoblast differentiation of mBMSCs

**Figure 3:**
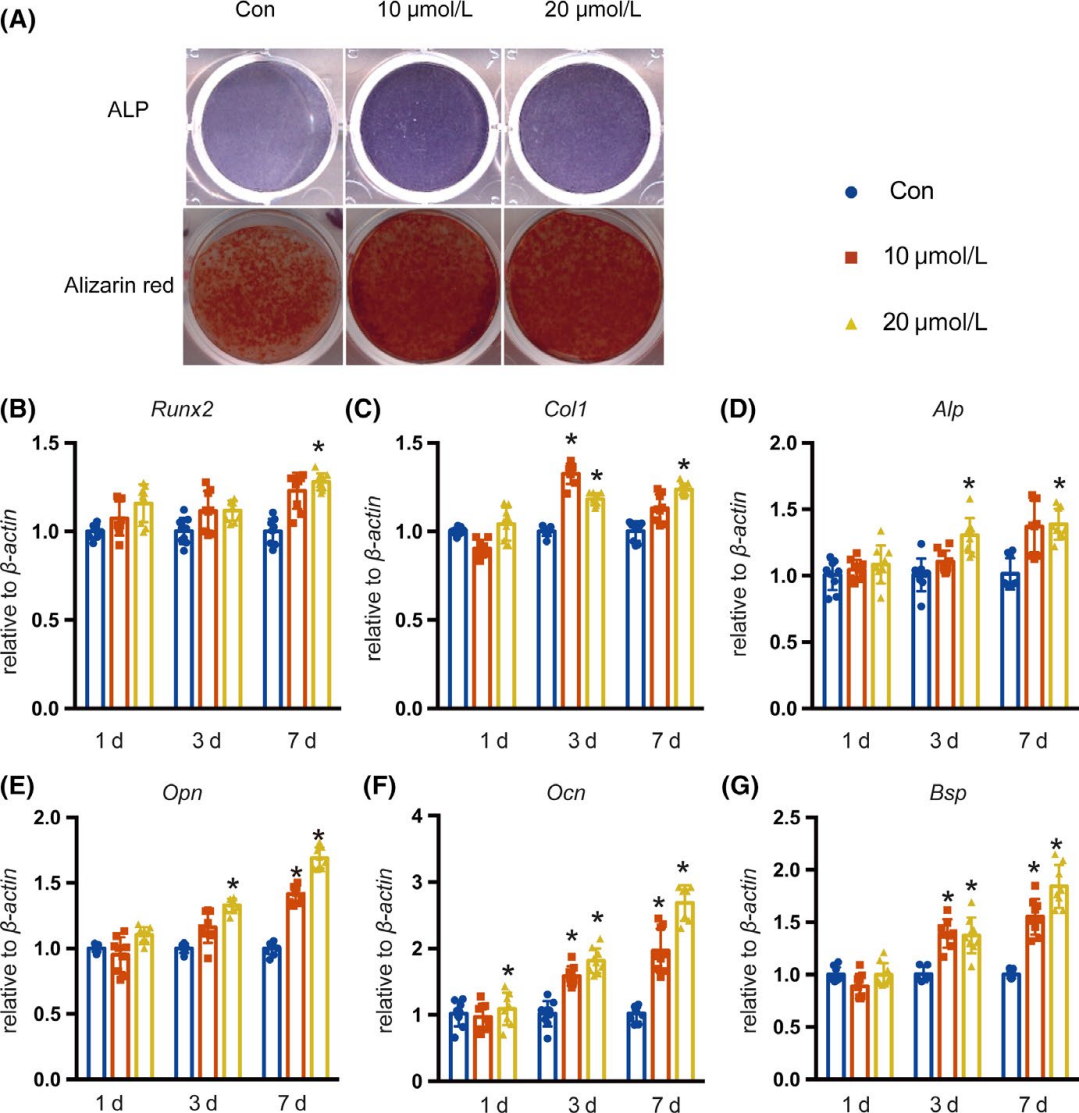
Effects of icariin on the osteoblastic differentiation of mBMSCs. A, ALP staining of mBMSCs after 7 days of treatment with icariin in osteogenic induction medium. Alizarin red staining of mBMSCs after 14 days of treatment with icariin in osteogenic induction medium. B‐G, Real‐time PCR analysis of *Runx2*, *Col I*, *Alp*, *Opn*, *Ocn* and *Bsp* mRNA in mBMSCs treated with icariin at concentrations of 10 or 20 μmol/L. Error bars represent mean ± SD. Significant differences are indicated by *P < .05; n = 9

We cultured mBMSCs in osteogenic induction medium with different concentrations of icariin and found that icariin promoted osteoblast differentiation, as indicated by a slight increase of alkaline phosphatase (ALP) activity and a dramatic increase of calcified nodule formation (Figure 3A). We next assessed the expression of the early osteogenic marker genes Runt‐related transcription factor‐2 (*Runx2*), collagen 1α1 (*Col1*) and *Alp*, and the late osteogenesis marker genes osteopontin (*Opn*), osteocalcin (*Ocn*), and bone sialoprotein (*Bsp*) by quantitative RT‐PCR. We found slight increases in the early marker genes, while the late osteogenesis marker genes showed changes of more than 2‐ to 2.5‐fold (Figure 3B‐G). Taken together, these data indicated that icariin promoted osteoblastic differentiation of mBMSCs, but its mechanism remained undiscovered.

### 3.4 STAT3 signalling is critical for icariin‐promoted osteoblast differentiation

**Figure 4:**
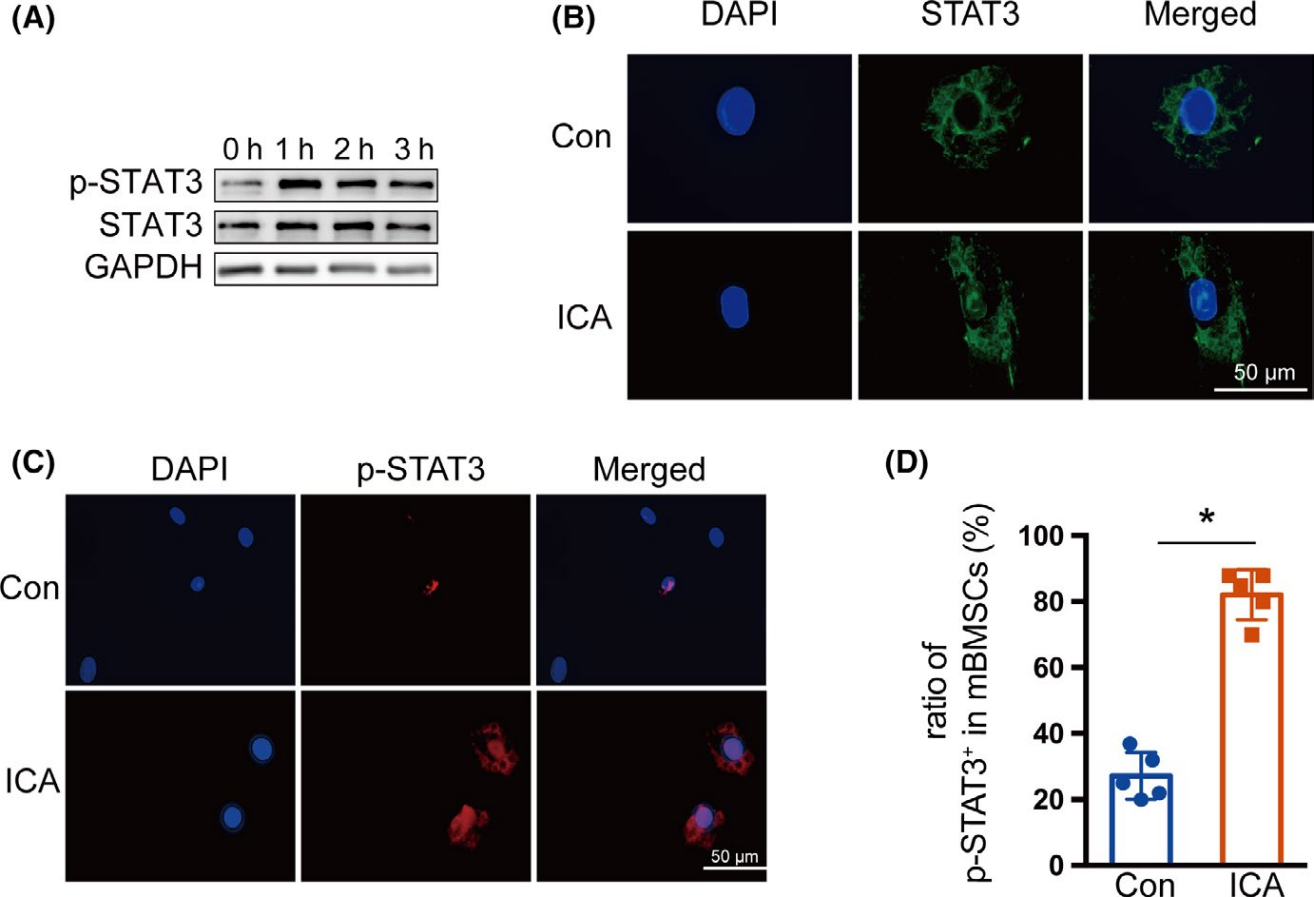
Effects of icariin on STAT3 activity. A, Protein levels of STAT3 and phosphorylation of STAT3 were examined by Western blotting after exposure to icariin (20 μmol/L) at the indicated time points. B, Immunofluorescence staining of STAT3 (green) in control and icariin‐treated mBMSCs for 1 h. C, Immunofluorescence staining of p‐STAT3 (red) in control and icariin‐treated mBMSCs for 1 h. D, The ratio of p‐STAT3^+^ cells in control and icariin‐treated groups. Error bars represent mean ± SD, *P < .05, n = 3

**Figure 5:**
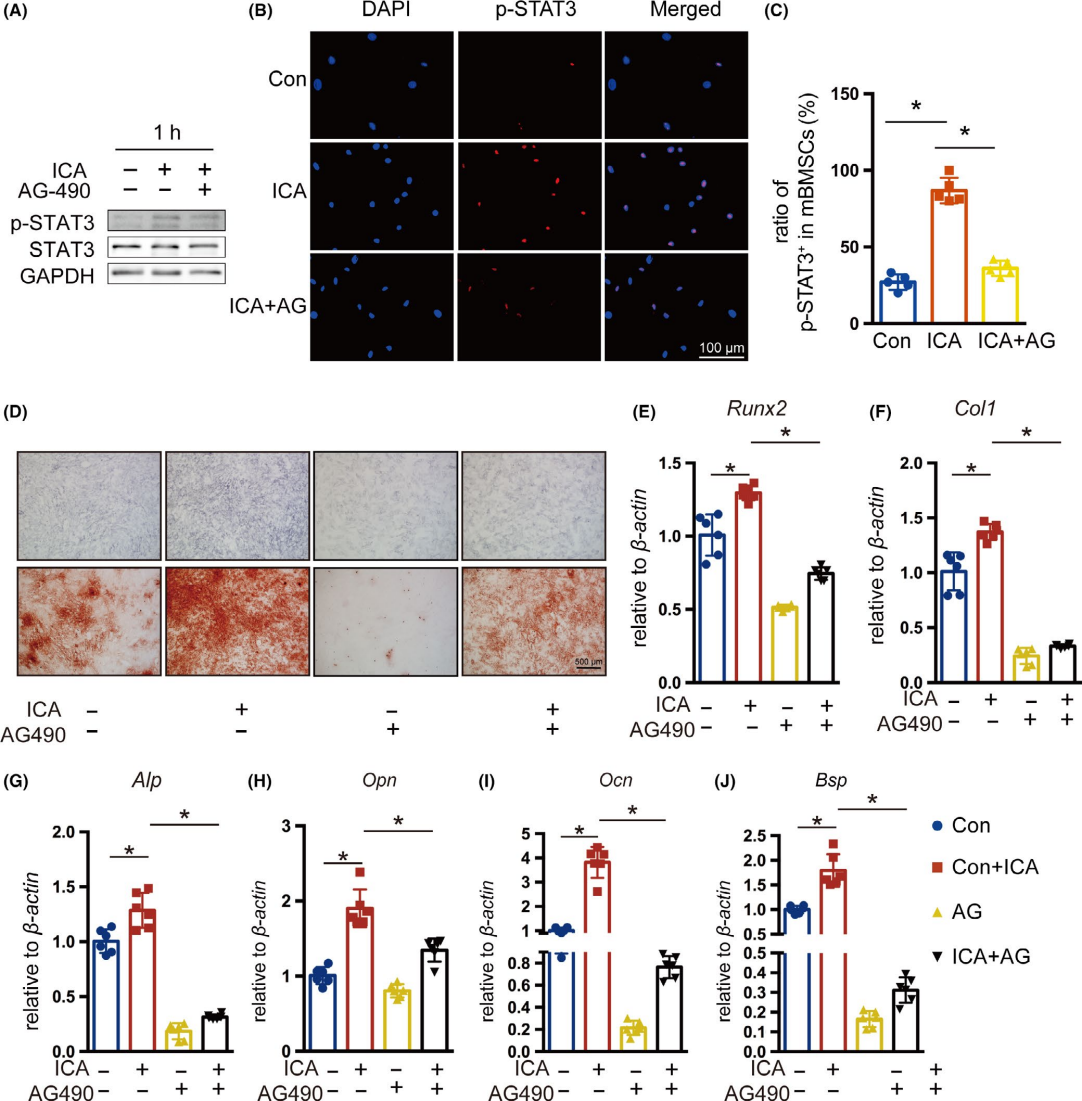
Involvement of the STAT3 signalling pathway in icariin‐induced osteoblastic differentiation of mBMSCs. A, Protein levels of STAT3 and phosphorylation of STAT3 in mBMSCs after treatment with AG490 for one hour, visualized by Western blotting. B, Immunofluorescence staining of p‐STAT3 (red) in control, icariin‐ and AG490‐treated mBMSCs. C, The proportion of p‐STAT3^+^ cells in control, icariin‐ and AG490‐treated groups. Error bars represent mean ± SD, *P < .05, n = 3. D, ALP staining and alizarin red staining of mBMSCs after treatment with AG490 on day 7 and day 14. E‐J, Real‐time PCR analysis of *Runx2*, *Col I*, *Alp*, *Opn*, *Ocn* and *Bsp* mRNA in mBMSCs treated with AG490 on day 7. Error bars represent mean ± SD. Significant differences are indicated by *P < .05; n = 6

Since icariin could regulate the activation of STAT3 which plays an important role in osteoblast differentiation, we analysed the protein levels of p‐STAT3 in mBMSCs that were treated with 20 μmol/L icariin after osteogenic induction for 1 day (Figure 4A). The results indicated that icariin treatment led to an increase in the phosphorylation level of STAT3 in comparison with the control group. Immunofluorescence staining suggested that icariin could promote nuclear translocation of STAT3 (Figure 4B) and also showed an increased number of p‐STAT3‐positive cells among icariin‐treated mBMSCs (Figure 4C,D). These results showed that icariin could regulate the activation of STAT3. Furthermore, we treated mandibular BMSCs with AG490 to inhibit the activation of STAT3 in mBMSCs at indicated time (Figure 5A). Immunofluorescence staining also indicated a decreased number of p‐STAT3‐positive cells among AG490‐treated mBMSCs (Figure 5B and C). ALP and alizarin red staining showed that AG490 inhibited icariin‐induced osteoblast differentiation, indicated by decreased ALP activity and ossification (Figure 5D). The icariin‐enhanced mRNA expression of the osteogenesis‐related genes *Runx2*, *Col1*, *Alp*, *Opn*, *Ocn* and *Bsp* was also repressed in the AG490‐treated group on day 7 (Figure 5E‐J). These results suggested that the STAT3 signalling pathway was critical for icariin‐promoted osteoblastic differentiation of mBMSCs.

### 3.5 Icariin regulates osteocalcin transcription through STAT3

**Figure 6:**
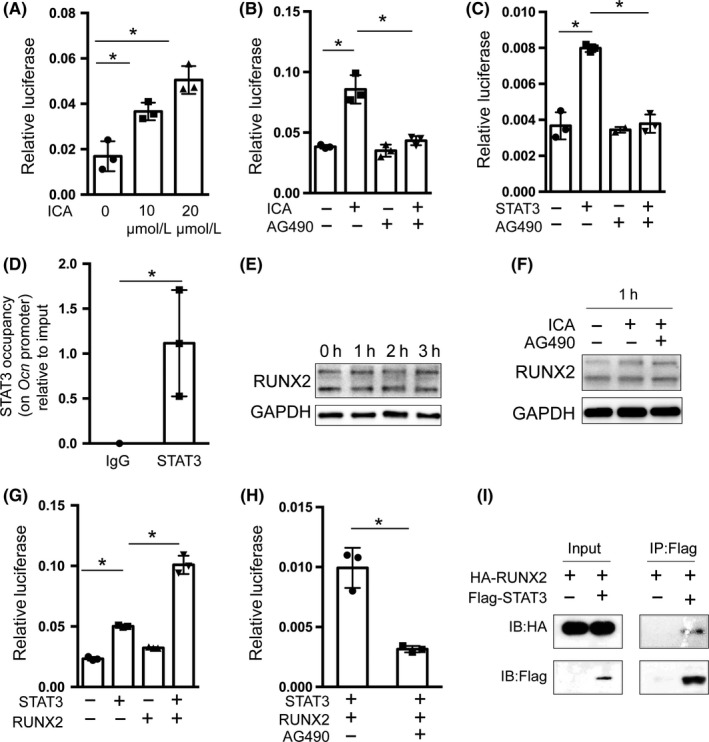
Icariin regulated *Ocn* transcription through STAT3 in cooperation with Runx2. A, *Ocn* promoter‐driven luciferase reporter and pRL‐TK were co‐transfected into HEK 293T cells with or without icariin using Lipofectamine 2000. After 48 h, luminescent signals were detected to represent reporter activity. B, HEK 293T cells were transfected with OG2‐Luc and pRL‐TK, and then treated with or without icariin and AG490. C, HEK 293T cells were transfected with OG2‐Luc, pRL‐TK, and Flag‐stat3, and then treated with or without AG490. D, Chromatin immunoprecipitation (ChIP)‐qPCR analysis of STAT3 in *Ocn* promoter in C_3_H_10_ cells. Immunoprecipitation was performed with anti‐STAT3, and IgG was used as a negative control. Precipitated DNA was detected by qPCR with specific primers. E‐F, Protein levels of Runx2 in mBMSCs were examined by Western blotting after exposure to icariin (20 μmol/L) or AG490 at the indicated time points. G, HEK 293T cells were transfected with OG2‐Luc, pRL‐TK and Flag‐stat3, together with or without HA‐Runx2. H, HEK 293T cells were transfected with OG2‐Luc, pRL‐TK, Flag‐stat3, and HA‐Runx2, and then treated with or without AG490. I, Co‐immunoprecipitation analysis of Flag‐STAT3 and HA‐Runx2 was performed in 293T cells. A Flag‐STAT3 expression plasmid was co‐transfected with HA‐Runx2 into 293T cells. Whole cell lysates (WCL) were used for immunoprecipitation (IP) and then immunoblotting (IB) with the indicated antibodies. IP products were detected by Western blotting with the indicated antibodies. Data represent means ± SD. *P < .05, **P < .01; n = 3

We next sought to further characterize the underlying molecular mechanism through which STAT3 regulates downstream osteogenesis‐related markers. Due to significant changes in the mRNA expression of late osteoblast maker genes, such as *Ocn*, we hypothesized that STAT3 regulates these genes directly. We first analysed the effects of icariin on *Ocn* transcriptional activity, using its promoter‐driven luciferase reporter. As shown in Figure 6A, the activity of the *Ocn* promoter was increased by different concentrations of icariin. Furthermore, AG490 blocked icariin‐driven *Ocn* promoter activity (Figure 6B). As shown in Figure 6C, we next found that STAT3 promoted *Ocn* transcription, which was also blocked by AG490. These results proved that icariin induces *Ocn* transcription through STAT3. Furthermore, we directly determined whether STAT3 could bind to *Ocn* promoter by using a ChIP assay. As shown in Figure 6D, STAT3 was enriched in the promoter of *Ocn*, indicating that STAT3 directly bound to the *Ocn* promoter to regulate *Ocn* gene expression. Meanwhile, it has previously been demonstrated that Runx2 is essential for the transcription of *Ocn* via binding to its promoter.^37^, ^38^ We previously found there was a slight increase of Runx2 mRNA in mBMSCs treated with icariin. Next, we found the protein level of Runx2 did not change in icariin‐treated mBMSCs with or without AG490 (Figure 6E and F). Hence, we assumed a possibility that STAT3 and Runx2 may functionally cooperate to regulate *Ocn* transcription. To test this hypothesis, we co‐transfected STAT3 and Runx2 into HEK 293T cells to analyse their effects on *Ocn* promoter activity. The results suggested that Runx2 promoted *Ocn* promoter activity, and combination with STAT3 could further enhance this effect (Figure 6G). Meanwhile, AG490 blocked the synergistic effects of STAT3 and Runx2 on *Ocn* promoter activity (Figure 6H). Furthermore, the protein interaction between STAT3 and Runx2 was demonstrated by the co‐immunoprecipitation (Co‐IP) assay (Figure 6I). These data suggested that STAT3 and Runx2 form a physical complex to regulate *Ocn* transcription, which may participate in icariin‐promoted osteoblast differentiation.

### 3.6 Icariin prevents oestrogen deficiency–induced alveolar bone loss

**Figure 7:**
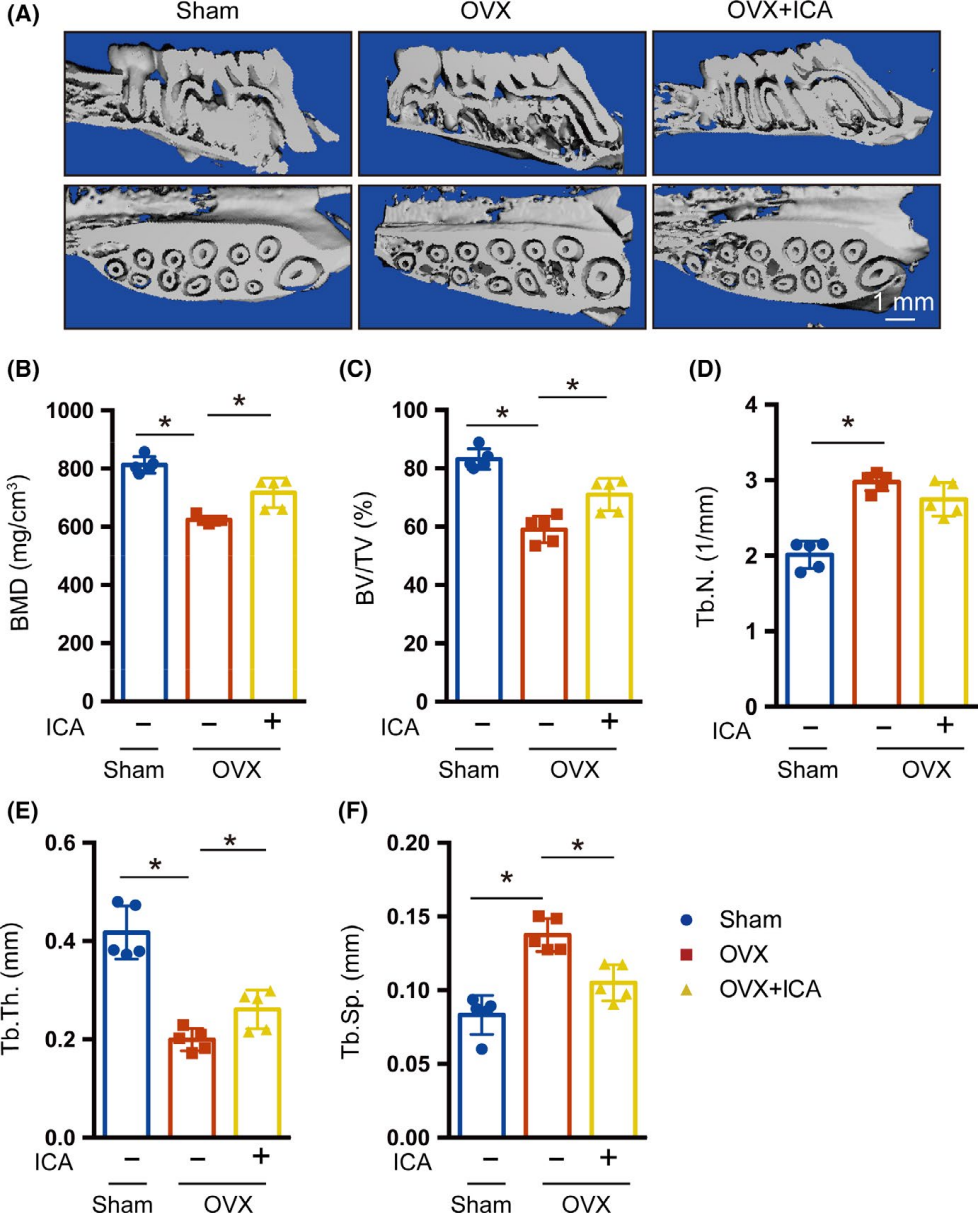
Icariin prevented oestrogen deficiency–induced alveolar bone loss. A, Representative images of changes in bone microarchitecture of the alveolar bone, analysed by micro‐CT analysis. Bar diagrams illustrate the microarchitectural parametric values of the alveolar bone. Parameters of TMD (B), BV/TV (C), Tb.N (D), Tb.Th. (E) and Tb.Sp (F) Error bars represent mean ± SD. Significant differences are indicated by *P < .05; n = 5

Next, we analysed the effects of icariin on oestrogen deficiency‐induced alveolar bone loss in an ovariectomized rat model. Micro‐CT was used to identify the effects of icariin on alveolar bone mass and quality in OVX rats. As shown in Figure 7A, the porotic changes in the maxillary alveolar bone of OVX rats were improved by icariin treatment, resulting in regeneration of more plate‐like trabeculae. Statistical analysis of the microarchitectural parameters showed that the OVX + icariin group had increased TMD, BV/TV and Tb.Th, and decreased Tb.Sp, but no difference in Tb.N when compared with the OVX group (Figure 7B‐F). These results indicate that icariin prevents the alveolar bone loss and microarchitectural deterioration induced by oestrogen deficiency.

### 3.7 Icariin promotes bone formation in the alveolar bone of OVX rats

**Figure 8:**
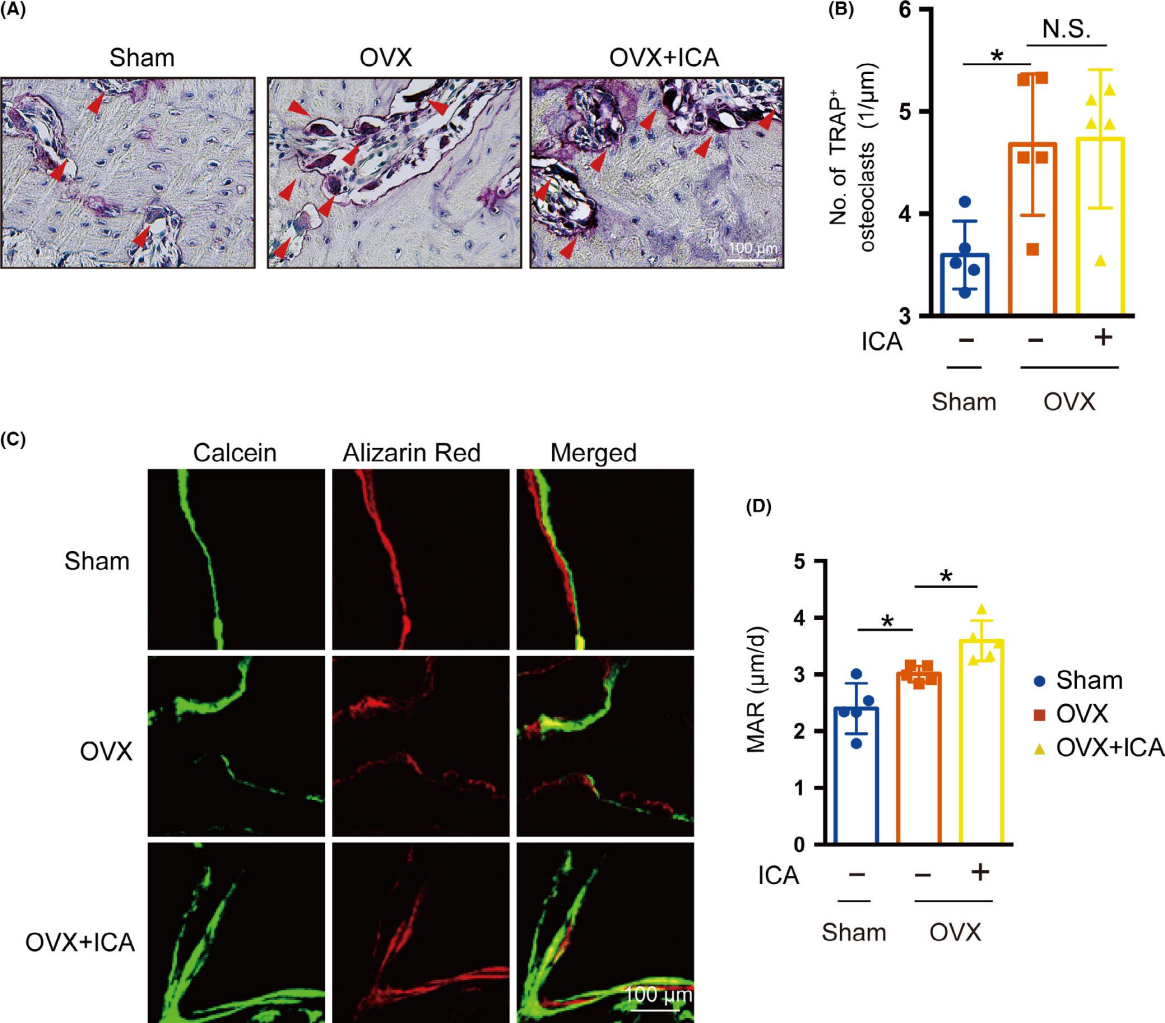
Icariin promoted bone formation in OVX rats. A, TRAP staining showed wine‐red multinuclear osteoclasts lining the margin of the trabeculae (red arrowhead in A). B, The number of osteoclasts in the OVX group was counted. C, Osteogenic activity in the maxilla of 12‐week‐old rats, shown by calcein and alizarin red double staining. Eleven‐week‐old rats received an intraperitoneal injection of 20 mg/kg calcein on day 0 and 40 mg/kg alizarin red on day 4, and then were sacrificed on day 7. The top panel shows a representative image of the SHAM group, the middle panel a representative image of the OVX group and the bottom panel a representative image of the OVX + ICA group. D, Mineral apposition rate (MAR) evaluated by histomorphometric analysis. Error bars represent mean ± SD, *P < .05; n = 5

The prevention of bone loss can be the result of increased bone formation, decreased bone resorption or a combination of both effects. To determine which mechanism was responsible for the prevention of alveolar bone loss in OVX rats, we first analysed bone resorption through TRAP staining. As shown in Figure 8A and B, the number of TRAP‐positive osteoclasts increased in the OVX group in comparison with the SHAM group, but there was no difference between OVX and icariin‐treated OVX rats, which suggested that the influences of icariin on alveolar bone may not depending on inhibiting bone resorption in OVX rats. Next, we analysed bone formation by calcein and alizarin red double labelling. Our results indicated that icariin promoted bone formation, as suggested by increased mineral apposition rate (MAR) compared with the OVX group (Figure 8C and D). These findings suggested that icariin prevents oestrogen deficiency–induced alveolar bone loss via promotion of bone formation, which was consistent with the finding that icariin promotes osteoblast differentiation of mBMSCs.

## 4 DISCUSSION

Osteoporosis, a disease characterized by reduced bone mass and increased skeletal fragility, affects about 10.3% of people around the world with the vast majority of cases occurring in post‐menopausal women, induced by ovarian oestrogen deficiency.^39^ Our previous studies revealed that oestrogen deficiency‐induced osteoporosis is also manifested in alveolar bone, causing deterioration of trabecular structure and loss of bone mass.^6^ Alveolar bone is the irregular protuberance of the jaw bone, which encompasses the roots of the teeth. Oestrogen deficiency–induced alveolar bone loss can lead to tooth loss and failures of dental treatment, ultimately causing a decrease in the quality of life. However, current treatment and prevention of osteoporosis do not pay attention to alveolar bone loss, and the problem is further complicated by the fact that the most common treatment agents, bisphosphonates, may cause serious adverse reactions in the jaw, such as osteonecrosis.^40^, ^41^ Thus, identifying therapeutic agents to alleviate osteoporosis of alveolar bone will have significant clinical value. In the present study, we found that icariin could prevent alveolar bone loss induced by oestrogen deficiency and thus could be used for the treatment of alveolar bone osteoporosis.

Researchers first established the key role of oestrogen in treating post‐menopausal osteoporosis. However, oestrogen treatment has since been found to have side effects including increased risk of cardiovascular events and breast cancer^16^; hence, the focus has gradually shifted to new drugs that are on the horizon, such as phytoestrogens. Icariin, one representative phytoestrogen, is a classic flavonoid extracted from *Herba epimedii* that has the potential to treat various diseases including heart disease, cancer and autoimmune disorders because of its similarities to oestrogen. Most importantly, it has been demonstrated that icariin can prevent and treat bone loss in different types of osteoporosis, demonstrated in model systems induced by oestrogen deficiency,^42^ chronic high‐dose alcohol^43^ or glucocorticoid,^44^ but most of the previous studies focused on the long bone system which differs from alveolar bone in terms of morphological features, metabolic rate and pharmaceutical reaction. Hence, we wondered what effect icariin would have on alveolar bone. Consequently, in the present study, we examined the effect of icariin on mandibular BMSCs and OVX‐induced alveolar bone loss.

We determined the optimal concentration of icariin to be 10‐20 μmol/L, through CCK‐8 and apoptosis assays. Then, we found that icariin exhibited the potential to promote osteoblastic differentiation of mandibular BMSCs in vitro, as shown by increases in ALP activity and calcification and upregulation of mRNA expression of osteoblast marker genes. These data are consistent with previous research in femoral BMSCs. However, the underlying mechanism remained undiscovered.

**Figure 9:**
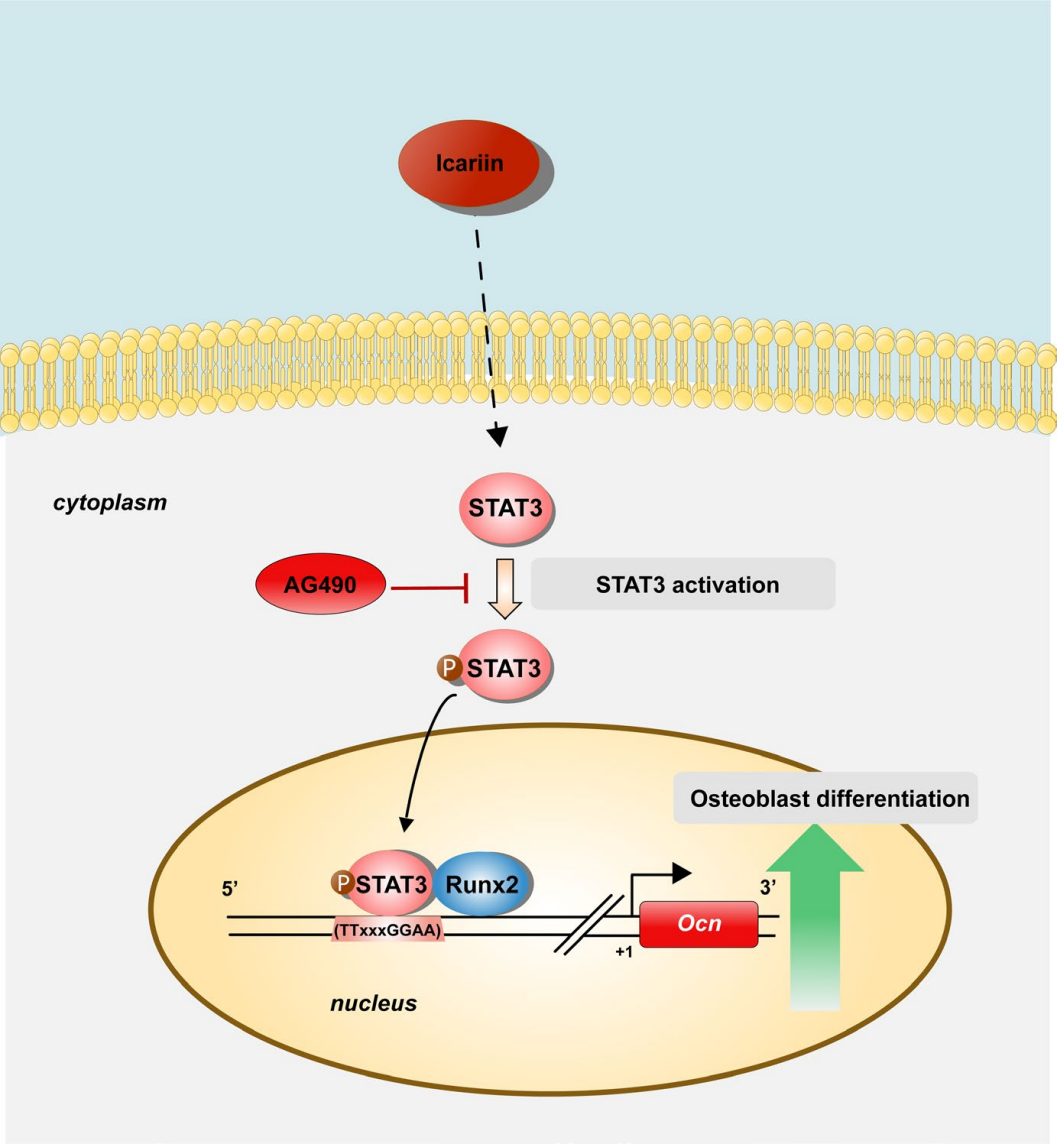
Schematic diagram showing icariin effects on promoting osteoblast differentiation of mandibular BMSCs through regulation of STAT3 activity. Icariin treatment induced the phosphorylation/ activation of STAT3. The phosphorylated form of STAT3 then promoted the transcription of *Ocn* through binding to its promoter in cooperation with Runx2

Multiple mechanisms and targets are involved in the therapeutic effects of icariin, such as NF‐κB and Erk‐p38‐JNK, TLRs and STATs. Among these, STAT3 aroused our interest due to its role in the bone system. STAT3 is a key signal transduction protein that integrates signalling by numerous growth factors, cytokines and oncoproteins. Once activated, STAT3 phosphorylates and translocates to the nucleus where it binds to target‐gene promoter sequences and induces gene expression.^45^ We found that the phosphorylation level of STAT3 was increased in icariin‐treated mBMSCs, while blocking of STAT3 activation with AG490 repressed icariin‐driven osteoblast differentiation. These data suggested that icariin could promote osteoblast differentiation though STAT3 signalling, which has not been reported before. To further study the molecular mechanism, we analysed the role of icariin on *Ocn* promoter activity via a luciferase reporter system because of the obvious increase of *Ocn* expression. Our results confirmed that icariin promoted *Ocn* transcription activity through STAT3 signalling. ChIP assay confirmed that STAT3 could directly bind to the Ocn promoter. Another critical transcription factor, Runx2, is essential for Ocn transcription by directly binding to and activating its promoter.^46^, ^47^ Further, we also found that there was only a slight change of Runx2 mRNA. Hence, we wondered whether STAT3 could interact with Runx2 to regulate *Ocn* transcriptional activity, which has not been reported before. Our results indicated that STAT3 and Runx2 exhibited a positive synergistic effect on Ocn promoter activity. Co‐IP assay further confirmed the protein interaction of STAT3 and Runx2. In summary, we proved for the first time that STAT3 and Runx2 can form a physical complex to promote *Ocn* transcription, which may participate in icariin‐promoted osteoblast differentiation (Figure 9).

In an OVX rat model, we found that icariin prevented oestrogen deficiency–induced alveolar bone loss, as indicated by increases of TMD, BV/TV and Tb.Th in icariin‐treated OVX rats in comparison with the OVX group. These data indicated that icariin could partly prevent oestrogen deficiency–induced alveolar bone loss through promotion of bone formation.

In conclusion, we demonstrate for the first time, to our knowledge, that icariin promotes osteoblastic differentiation of mandibular BMSCs through STAT3 signalling. In addition, we show that icariin treatment prevents deficiency‐induced alveolar bone loss by promoting bone formation. Based on these results, we propose that icariin could be a promising candidate treatment for oestrogen deficiency–induced alveolar bone loss, although preclinical pharmacological studies will be required.

## CONFLICT OF INTEREST

The authors declare that they have no conflicts of interest with respect to the contents of this article.

## AUTHOR CONTRIBUTIONS

Qinggang Dai and Lingyong Jiang designed the research; Hongyuan Xu analysed the data; Hongyuan Xu, Siru Zhou, Ranyi Qu, Yiling Yang, Xinyi Gong, Yueyang Hong and Anting Jin performed the research; Xiangru Huang, Hongyuan Xu, Siru Zhou and Ranyi Qu wrote the paper; and Qinggang Dai and Lingyong Jiang contributed new reagents or analytic tools.

## Data Availability

All data generated or analysed during this study are available in this article.
